# Examining Gravettian and Magdalenian mobility and technological organization with IR spectroscopy

**DOI:** 10.1038/s41598-024-84302-6

**Published:** 2025-01-14

**Authors:** Benjamin Schürch, Nicholas J. Conard, Patrick Schmidt

**Affiliations:** 1 Department of Early Prehistory and Quaternary Ecology, Institute of Prehistory, Early History and Medieval Archeology, University of Tübingen, Tübingen, Germany; 2 Senckenberg Centre for Human Evolution and Paleoenvironment, University of Tübingen, Tubingen, Germany; 3 Applied Mineralogy, Department of Geosciences, Eberhard Karls University of Tübingen, Tübingen, Germany

**Keywords:** Provenance analysis, Raw material procurement, Hunter-gatherer mobility, Gravettian, Magdalenian, Archaeology, Cultural evolution

## Abstract

Archaeologists can use the provenance of lithic raw materials to examine the movements, territories, and settlement dynamics of hunter-gatherers. Several studies have used macroscopic analyses to propose the long-distance transport of raw material during the Gravettian and the Magdalenian of the Swabian Jura in Central Europe. Until now hypotheses about raw material transport in this region were not based on reproducible analyses. This study aims to test some of the hypotheses about the origins of lithic raw materials during the Gravettian and Magdalenian, using infrared spectroscopic measurements. These analyses are based on differences and similarities in the mineralogy and crystallography of rocks. Using this method, we test for long-distance raw-material transport between the sites of the Swabian Jura and the Freiburg basin, 200 km to the south-west, and the region of the Altmühl Valley, 150 km to the north-east. For this, we created a reference database of 114 lithic raw material outcrops from Southern Germany and compared these specimens with artifacts from eleven archeological sites. Our study reconstructs the raw-material procurement and transport during the Gravettian and Magdalenian and reveals settlement patterns and territories that span over more than 300 km in Central Germany.

**Supplementary Information:**

The online version contains supplementary material available at 10.1038/s41598-024-84302-6.

## Introduction

**Fig. 1:**
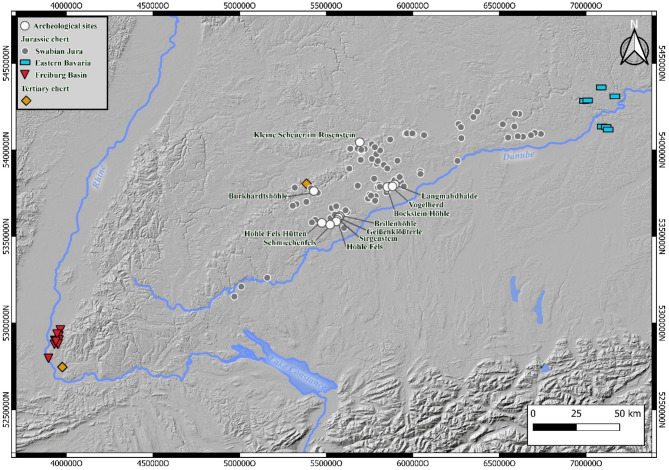
Overview map over Southern Germany, the sites addressed in this study and the sampled raw material outcrops (Basemap: © European Union^85^; map generated by B. Schürch using QGIS Geographic Information System v.3.16 (http://www.qgis.org)).

Raw material studies are often-used for reconstructing settlement dynamics during the Paleolithic^1–6^. Understanding transitions in raw material sourcing behaviors can reveal differences in land use. The results obtained by lithic raw material analyses, however, are only meaningful, if they are not affected by observer bias. This is rarely the case in Central Europe and especially in Southern Germany because raw material is mainly analyzed using macroscopic criteria^4,6–8^. Also, the few available empirical studies that were conducted on raw-materials in Southern Germany have either been able to provide limited insight into raw material use, or they have addressed specific research questions^9–12^. For this purpose, we created a reference collection of the major raw materials from Southern Germany. These are mostly Jurassic and Tertiary cherts (Fig. 1). To compare this reference collection with artifacts from different sites of the Swabian Jura, we use a recently proposed method based on reflectance infrared (IR) spectroscopy (see for ex^5^). Recent studies have applied this to contexts in Southern Germany^10^, France^13^, Poland^14^ and North America^5,15–17^ and their first results are promising. The method, initially proposed by Parish^5,15,17^, is based on non-destructive measurements of IR reflectance spectra on the surface of artifacts^10,14,15,17–21^. The spectra obtained contain information about the mineralogical content of the samples and several crystallographic parameters, such as crystal size and orientation or disorder-related lattice parameters^10^. Applying this technique to raw materials in Southern Germany should provide information on the raw-material procurement of human groups in the Gravettian and Magdalenian. For this purpose, we chose archaeological sites in the Swabian Jura, a region with an exceptionally rich record of Paleolithic sites that have been intensively studied since the late 19th century^22,23^.

We also assessed typological data on the studied archeological artifacts to investigate if it were tools or raw materials that were transported. Comparing the Gravettian and Magdalenian land use and artifact types that were transported can be informative on possible economic or cultural differences. Both technocomplexes are separated by the last glacial maximum. The Gravettian in the Swabian Jura ended at around 28,000 cal BP and the region was mostly uninhabited until occupation intensified in the Magdalenian at approximately 16,500 cal BP^24–28^.

### Investigated hypotheses

Our study is based on testing the claims of several macroscopic analyses^6,8,24,29–31^, namely long-distance connections between the sites of the Swabian Jura, the regions of the Freiburg basin (approx. 200 km distance) and the Franconian Jura (approx. 150 km distance)^24,25,29–37^. Additionally, we tested possible raw-material connections between the Swabian Jura sites and the Tertiary chert outcrops in the regions of the Randecker Maar and Tüllinger Berg^34,38^. The raw material outcrop of Tertiary chert at the Randecker Maar at the northern edge of the Swabian Jura was proposed to have been of great importance during the Magdalenian in southwestern Germany^33,34^. We further tested whether the same was already true in the Gravettian.

The best summary on raw material exploitation and long-distance raw material transport for the sites in the Swabian Jura is provided by Burkert^6^. The hypotheses proposed are largely supported by more recent macroscopic analyses, which, with a few exceptions^39^, refer to individual sites^24,25,30,31^. The hypotheses formulated by Burkert are as follows^6^:

- During the Magdalenian and the Gravettian, non-local raw materials were mainly sourced along the axis of the Danube.
- In the Gravettian there were already raw material connections to the Franconian Jura (called Jurassic chert Eastern Bavaria in this study) and the Freiburg basin (Burkert called the raw material “Blutjaspis”, this raw material is called Jurassic chert Freiburg basin in this study).
- The Gravettian sites have the same raw material catchment areas as the Magdalenian sites.
- The proportions of raw material from the Franconian Jura and the Randecker Maar (called Tertiary chert in this study) increase in the Magdalenian compared to the Gravettian.

## Results

**Fig. 2:**
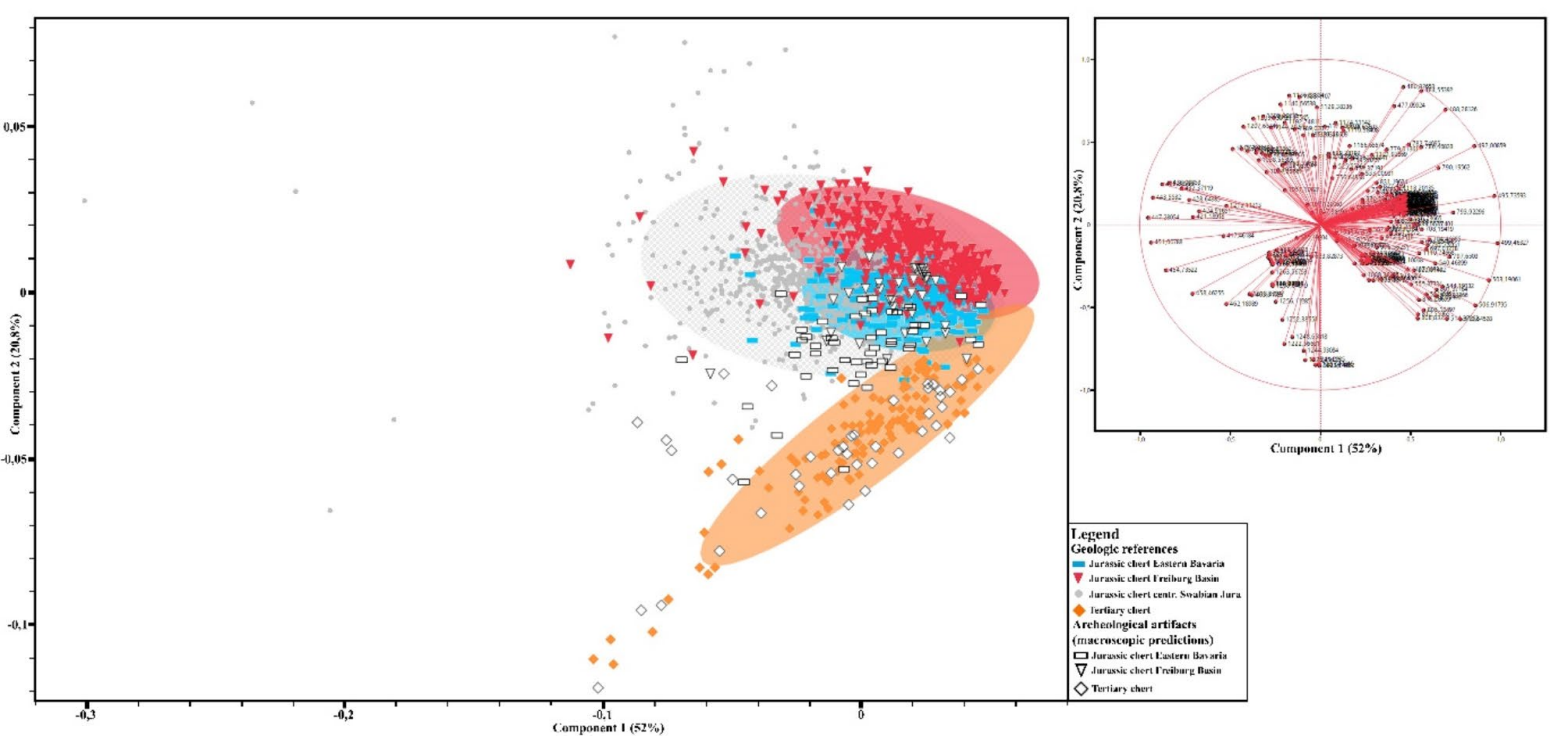
PCA scatter plot (covariance matrix) resulting from the first derivative data of all 243 variables of the spectra (left) and the loading plot showing the direction of different variables (in our case the intensities in the 1st derivative spectrum at different wave numbers) in the PCA biplot (top right). The archaeological samples are shown in the same color coding as in the tables above, Tertiary chert is orange-colored diamonds, Jurassic chert of the central Swabian Jura are grey points, Jurassic chert of Eastern Bavaria are blue rectangles, Jurassic chert of the Freiburg basin are red triangles. Ellipses show the 95% confidence intervals.

The reflectance spectra of the geological references and archeological samples were converted into first derivative spectra to minimize the effect of overall intensity differences in the spectra of different samples. This derivative data is representative of band shape and the presence/absence of bands in the original reflectance spectra. All intensities of these first derivative spectra between 1300 and 400 cm^− 1^ were used as variables, and their variance was compared using principal component analysis (PCA). The PCA plot resulting from our FTIR-analysis shows the variance between the reference raw materials and archaeological samples (Fig. 2). Tertiary chert is well differentiated from the three different variants of Jurassic chert in this plot. With few exceptions, archaeological samples macroscopically determined as Tertiary chert plot together with geological references of Tertiary chert. The differences between the other raw materials are less pronounced in this PCA plot. There are distinct but overlapping clusters of the three sampled variants of Jurassic chert. The overlap between Jurassic chert from the central Swabian Jura and eastern Bavaria is strongest. For archaeological samples, it can be challenging to determine their precise raw material categorization using a PCA. This difficulty arises when samples cannot be definitively assigned to a specific group or when they appear to fall between or within overlapping zones of different groups in the PCA.

To obtain more specific results, allowing us to assign artifacts to a raw material reference group, we constructed a neural model and performed a linear discriminant analysis (LDA) (Table 1). Both show high degrees of identifiability. We tested this degree of identifiability with accuracy tests. Our neural model analysis revealed a misclassification rate of 6.67% (*n* = 76 of 1139). LDA resulted in a misclassification rate of 13.51% (*n* = 220 of 1628). To test the accuracy of the neural model and the LDA, we performed accuracy tests with randomly selected subsamples of 30% of the geological references, removing their group assignment. These accuracy tests revealed a misclassification rate of the neural model of 10.4% (*n* = 51 of 489) and a rate of 20.8% for the LDA (*n* = 103 of 491) (Table 2). Misclassifications in the validation mostly appear between Jurassic chert of the central Swabian Jura and Jurassic chert of eastern Bavaria. For the neural model, this leads to a significant underestimation (70.8% are correctly classified) of the abundance of Jurassic chert of eastern Bavaria. They are often classified as being Jurassic chert of the central Swabian Jura. However, Jurassic chert of central Swabian Jura is correctly classified most of the time (93.7%). For the LDA, misclassification occurs for the Jurassic chert of the central Swabian Jura (only 66.7% are correctly classified) which is classified into the Jurassic chert of eastern Bavaria and into the Jurassic chert of the Freiburg basin. This leads to an overestimation of the Jurassic chert of eastern Bavaria and the Freiburg basin.

**Table 1:**
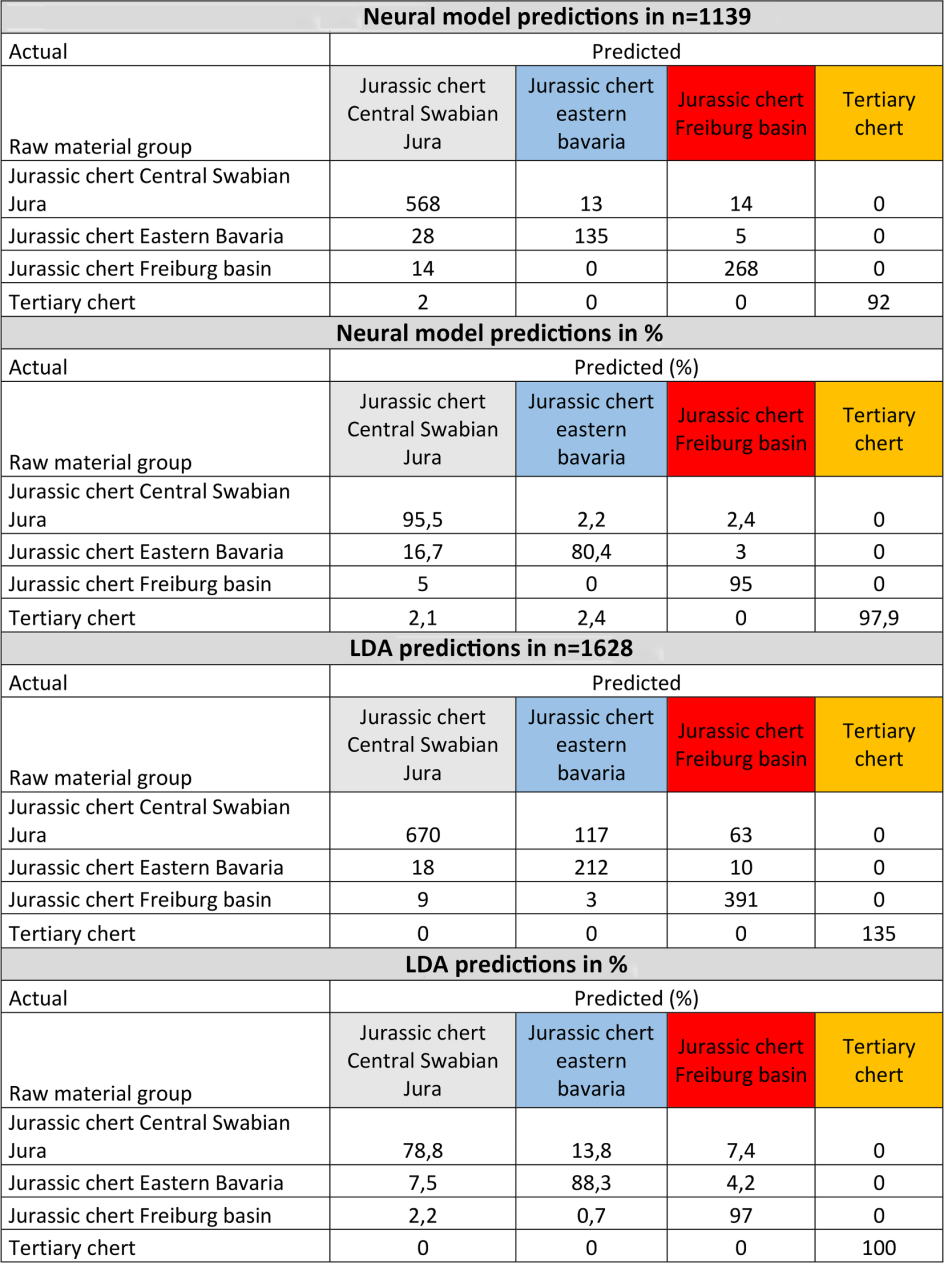
Neural model and LDA predictions (discriminant-scores) of the geological samples.

**Table 2:**
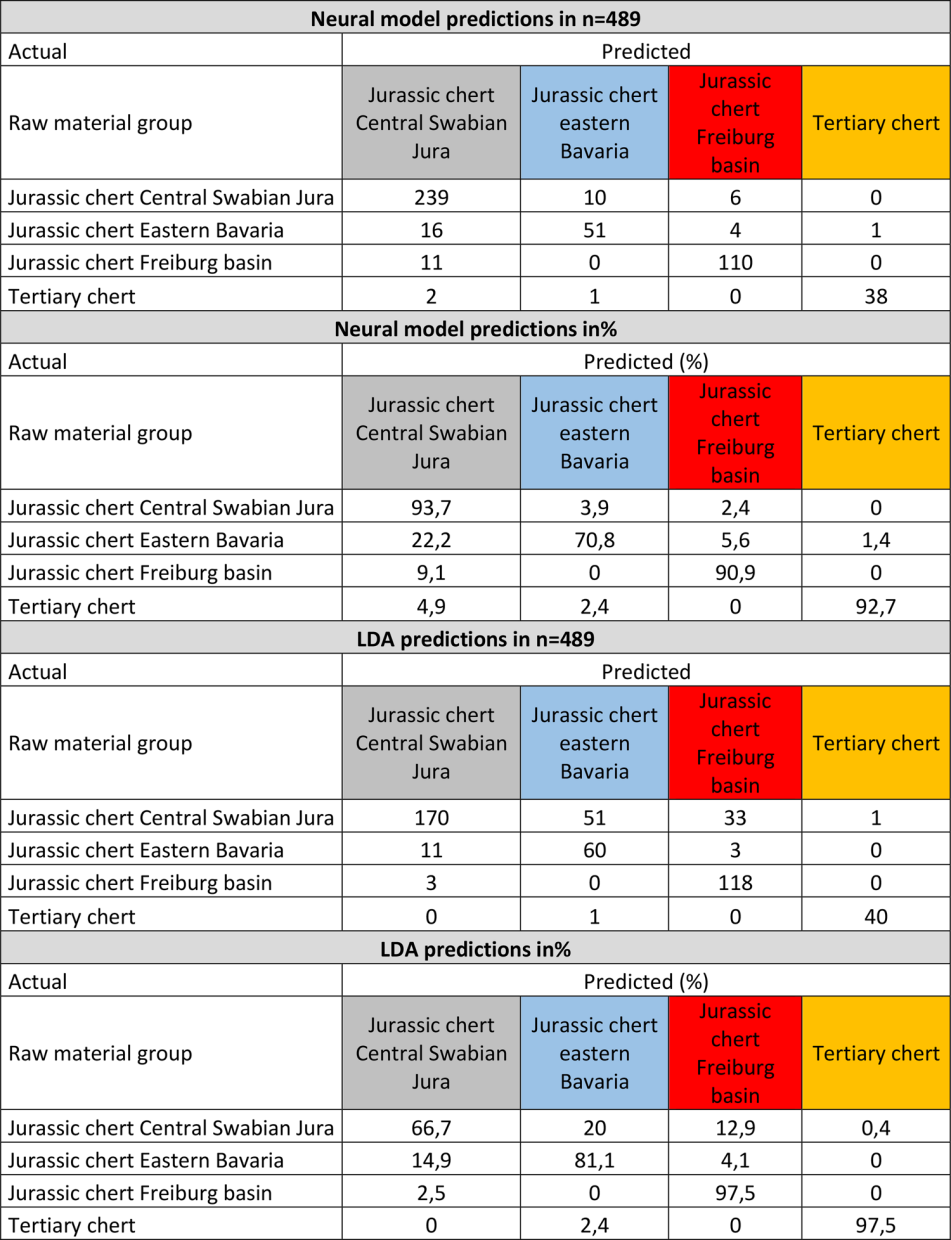
Summary of the neural model and LDA accuracy tests.

The predictions of our neural model (Tables 3 and 4) for archeological chert artifacts previously macroscopically assigned to Jurassic chert from eastern Bavaria are correct to 17.6%. Predictions of Jurassic chert of the Freiburg basin (75.8%) and Tertiary chert (82.5%) are better. The certainty of the predictions made for the artifacts macroscopically determined as Jurassic chert of eastern Bavaria are no greater than 57%. The predictions for the single artifacts macroscopically determined to be Jurassic chert of the Freiburg basin can be predicted with up to 100% certainty, and the Tertiary chert can be predicted up to 99% certainty. According to the LDA predictions (Tables 3 and 4) the artifacts, 47.1% of the macroscopically determined Jurassic cherts of eastern Bavaria are determined as such by the model. The numbers for the Jurassic chert of the Freiburg basin (84.8%) and the Tertiary chert (60%) are greater.

**Table 3:**
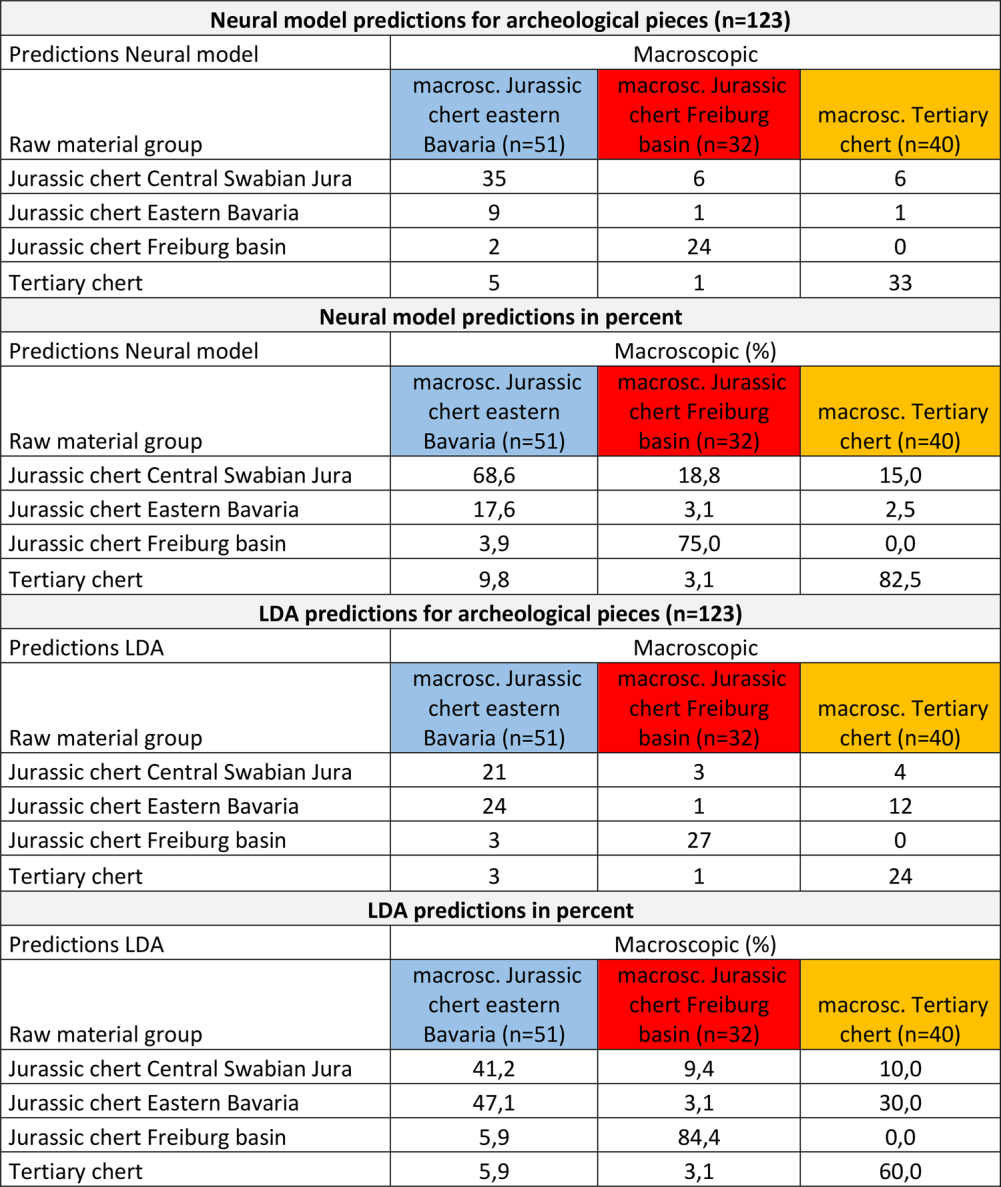
Predictions of the neural model and the LDA for the archaeological samples.

**Table 4:**
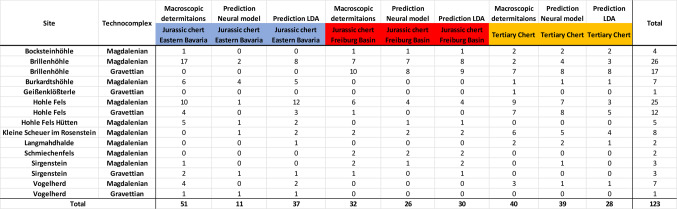
Overview of the archeological sites, technocomplexes and tested archeological pieces and the predictions of the neural model.

To assess whether the recorded signal of long-distance raw material transport can be attributed to the transport of tools or if raw material pieces were transported over a distance, we recorded typological data on the lithic artifacts (Table 5). Our data only includes artifacts assigned to specific outcrops by our neural model. We found that all three raw material groups were used for making tools in the Gravettian and the Magdalenian. Both formal tools (e.g. burins, end-scrapers, splintered pieces and backed artifacts) and informal tools (blade and blade fragments) of every raw material group are present. Furthermore, cores, refit sequences and products of core preparation and maintenance (e.g. decortification flakes, core tablets or crested blades) are also present.

**Table 5:**
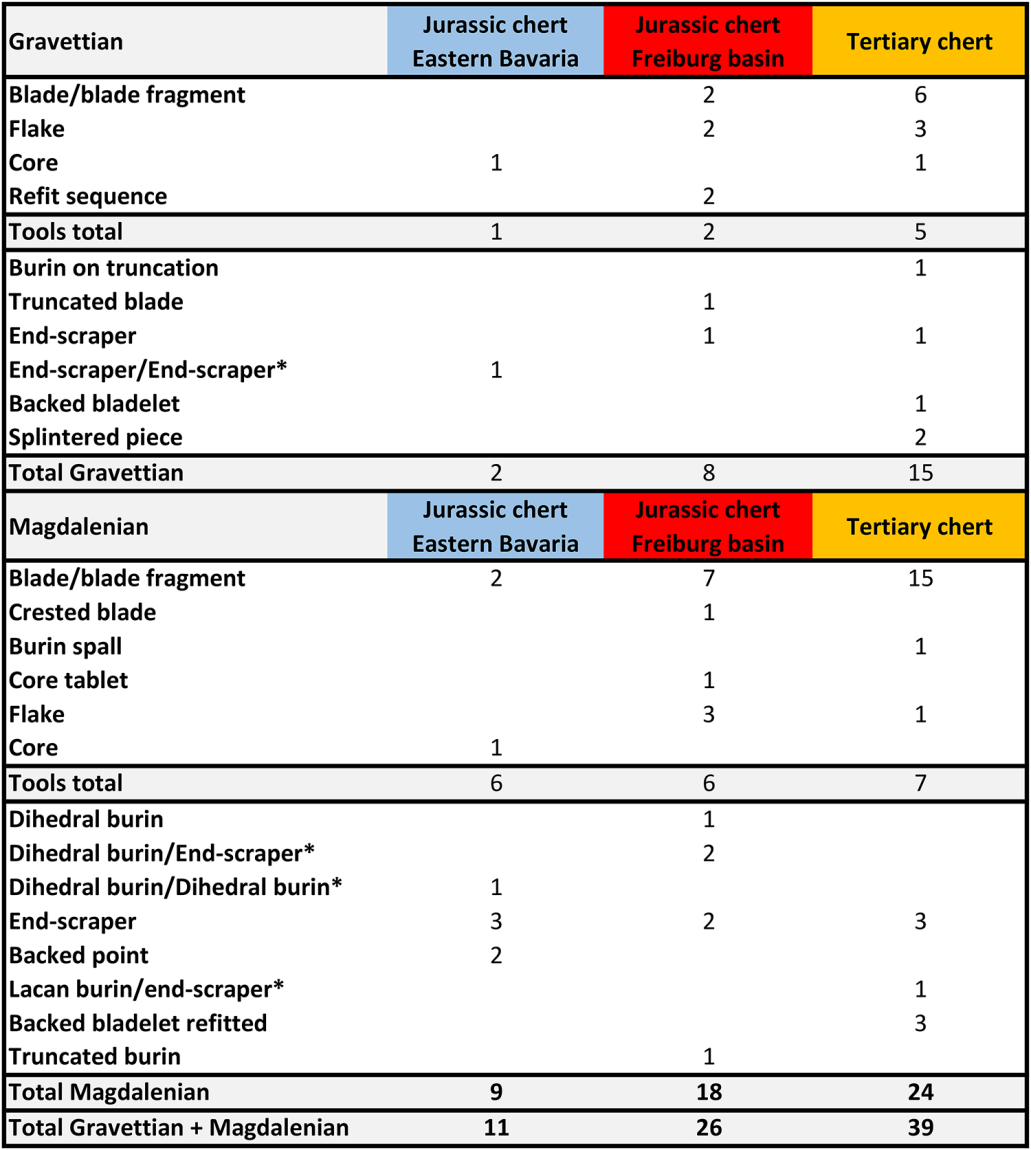
Overview of the archaeological artifacts and their typological identification, with macroscopic identification confirmed by the neural model (*combination tools with two modified ends).

## Discussion

### Accuracy of our models

The accuracy tests provide a good indication of the reliability of our models. The neural model seems to be better suited for predicting the geological origin of the raw materials. The margin of error of its accuracy test is 10.4%, which is significantly better than the 20.8% for the LDA. Our neural model is also better at not assigning the Jurassic chert of the central Swabian Jura to other groups of Jurassic cherts. The neural model has the greatest difficulty in identifying Jurassic chert of eastern Bavaria. Problems in distinguishing Jurassic chert of eastern Bavaria from that of the central Swabian Jura were already evident in the PCA, where large areas of the two groups overlap. These difficulties may be caused by the fact that both groups derive from the same geological formations (upper Malm) and are also macroscopically similar. Additionally, for individual artifacts, the neural model is better suited for making predictions. This is because the predictions for the Jurassic chert of eastern Bavaria usually have a lower prediction probability. The neural model thus reflects the difficulties of the overlap between the Jurassic chert of Swabia and Bavaria. The LDA does not seem to be as good for making such predictions, and it makes predictions with much greater certainty. This also applies to Jurassic chert of the central Swabian Jura, where the LDA revealed problems in accuracy tests. Therefore, the results of our LDA seem to be less reliable, whereas the results of the neural model appear to be better suited for making predictions.

**Fig. 4:**
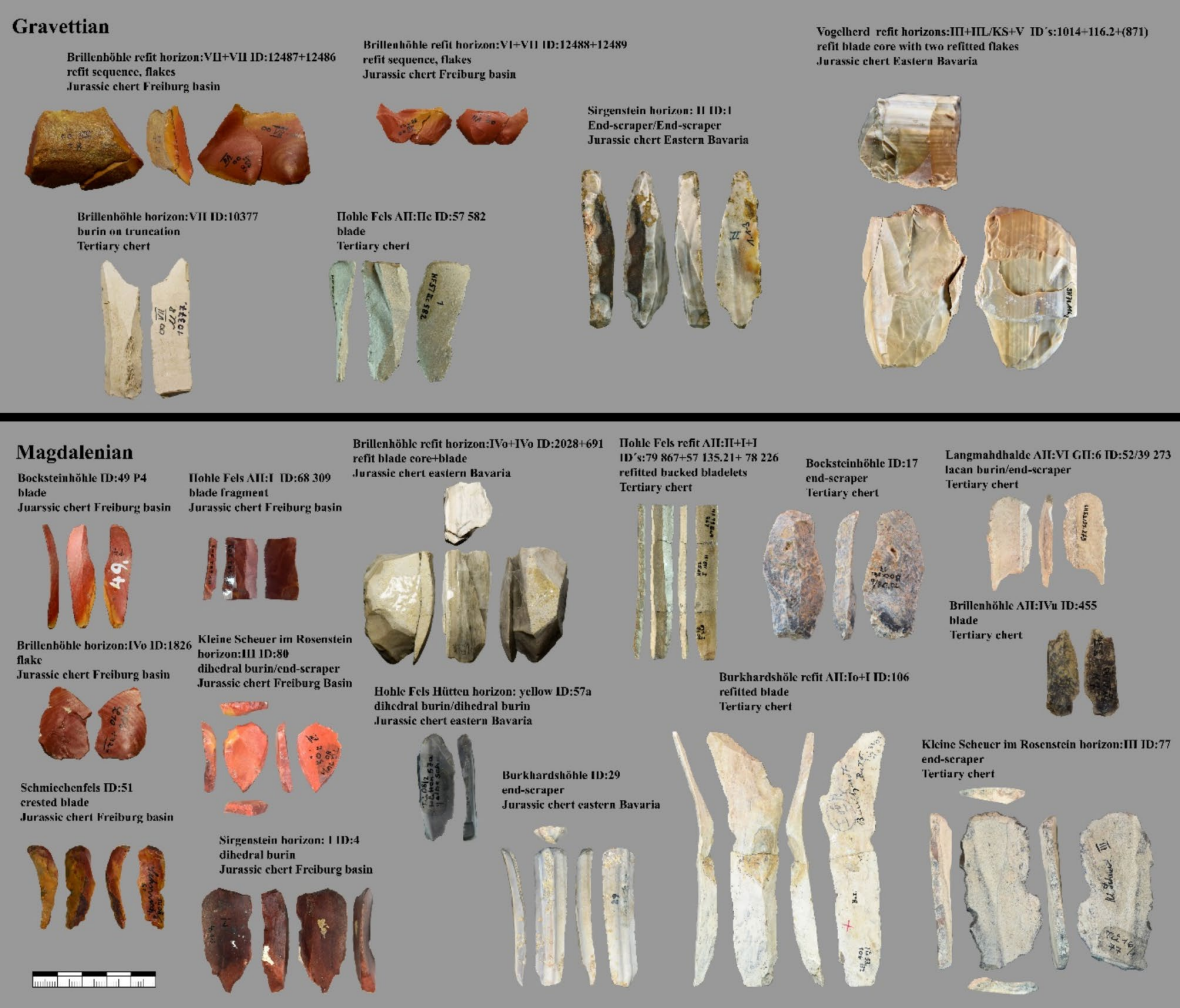
Archeological artifacts: the macroscopic determination could be confirmed for these artifacts by our analysis. The respective site, layer, ID, typological determination, and result of the neuronal model are listed individually for each artifact. Photos by B. Schürch.

Another factor that may have complicated predictions about the Jurassic chert of eastern Bavaria may be due to the variety of macroscopically determined archaeological artifacts of Jurassic chert of Bavaria. The most common macroscopic variety of archeological artifacts (the material is brown-grey striped parallel to the cortex and appears in plates, Fig. 4 core from Vogelherd) does not correspond to any variety of Jurassic chert found in outcrops known to us, neither from Bavaria nor from other areas. However, this material has a tabular characteristic, which suggests that it originated in Bavaria, only here the tabular varieties of Jurassic chert are found in Southern Germany^40^. Since our reference database for raw material outcrops from Bavaria is focused on deposits already used in the Paleolithic (such as Abensberg Arnhofen and Baiersdorf) and are mined in later periods^40,41^, this may further hamper the classification of archaeological artifacts determined to be Jurassic chert from Bavaria.

### Evidence for long-distance raw-material procurement

**Fig. 3:**
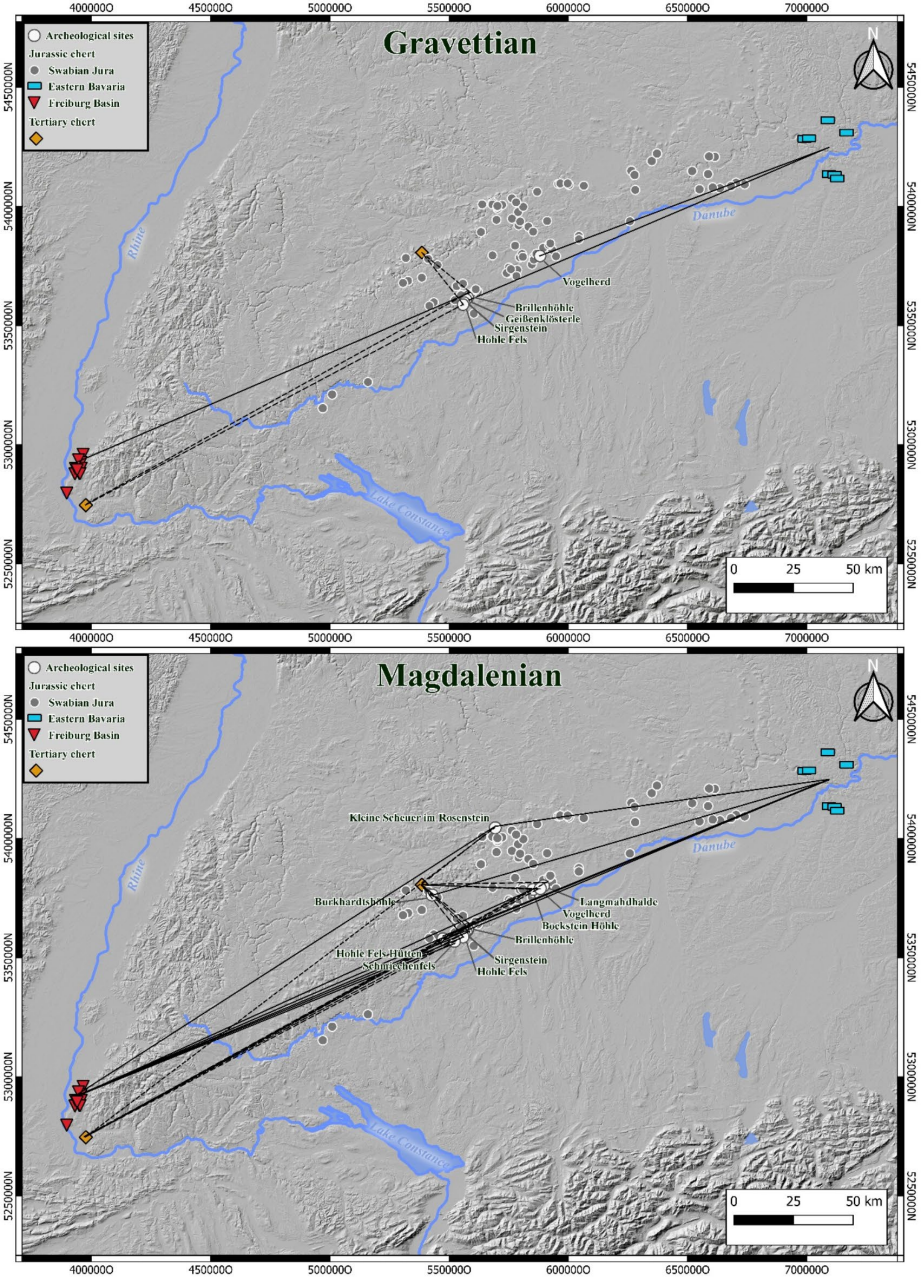
Long-distance raw material connection of archaeological sites in the Swabian Jura region during the Gravettian (top) and Magdalenian (bottom). (Basemap: © European Union^85^; map generated by B. Schürch using QGIS Geographic Information System v.3.16 (http://www.qgis.org))

Our results reveal patterns of long-distance raw material procurement in the Gravettian and Magdalenian (Fig. 3). Our analysis shows that, from the Gravettian onward, there were connections to all three tested raw material sources, namely to the Freiburg basin, 200 km south-west of the Swabian sites, to the region of the Altmühl Valley, 150 km to the north-east and to the outcrops of Tertiary chert. In the Magdalenian, these connections are more frequent. However, it must be pointed out that we only tested a sample of raw materials that were previously macroscopically determined. We did not test raw materials that may potentially show long-distance connections to the south or north in this study. These raw materials are radiolarite, quartz and quartzite. Testing connections to the south would be difficult, as the raw materials were mostly collected from river gravels from tributaries of the Danube and the Danube, as primary deposits were not accessible due to the Alpine glaciers. It would be possible to test the connections to the north, but our comparative database would need to be enlarged. However, when reconstructing mobility patterns connections to the south and north need to be considered as well (see supplementary Table 1).

Our data suggests that raw material procurement occurs both westward and eastward. The data therefore support Burkert’s hypothesis that non-local raw materials were mainly sourced along the axis of the Danube^6^. Future works should incorporate outcrops that are situated southwards or northwards as well, to enable more detailed insights into catchment areas. We were able to confirm that there are long-distance connections to the Franconian Jura and the Freiburg basin and connections to the outcrops of Tertiary chert. Our analysis showed that macroscopic analyses cannot necessarily be relied upon. Therefore, even if macroscopic analyses can be a good starting point, they need to be verified. We also found that the sites from the Gravettian show similar settlement patterns as the Magdalenian sites. Even if the quantity of connections differs. Burkert’s last hypothesis, that the amount of raw material from the Franconian Jura and the Randecker Maar (Tertiary chert) increases in the Magdalenian, must be viewed critically. Since we tested samples from the assemblages, this cannot be conclusively evaluated based on our data. However, if we rely on the available macroscopic analyses, the variation within the Magdalenian is large, which may be related to site function or the distance of the site to the outcrop. Therefore, this cannot be answered conclusively (Table 6).

**Table 6:**
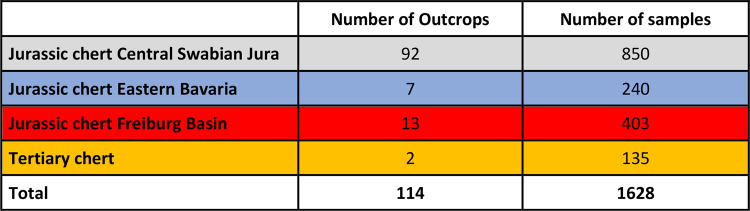
Number of outcrops and samples in the four sampled raw material groups.

**Table 7:**
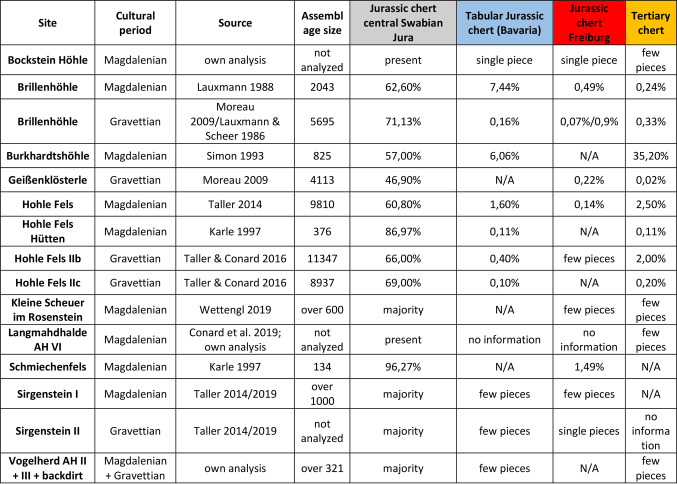
Overview of the Archeological sites with the corresponding layers, technocomplexes, sources of macroscopic determinations, assemblage size and percentages of the sampled raw materials in the archeological assemblages. For the complete raw materials data on the sites see supplementary (Supplementary Table 1).

These results of macroscopic analyses, however, can vary considerably depending on the analyst. One example are the analyses by Moreau and Lauxmann & Scheer, who reported different results for the proportion of Jurassic chert from Freiburg basin in the Gravettian of Brillenhöhle (Table 7, result Moreau: 0,07%; result Lauxmann & Scheer: 0,9%). This shows the variance between different analysts and the observer bias. This study is the first to provide unbiased data on long-distance lithic connections in the Magdalenian and Gravettian of southwestern Germany.

To interpret this intensification of long-distance raw material transport, we need to consider different factors, such as the number of sites, preservation and occupation intensity. The number of Gravettian sites in our study is significantly lower than that of Magdalenian sites. This is because the Gravettian is only well documented in the Ach Valley. Refits of Gravettian production sequence of artifacts from the Ach Valley of the sites Brillenhöhle, Sirgenstein, Hohle Fels and Geißenklösterle even prove the contemporaneous use of the caves at least for part of the deposits^29,30,36^.For the Lone Valley, recent studies have shown that Gravettian deposits are not or only sparsely preserved in the Lone Valley due to erosional events^28,42^. Other factors like the less precise excavation techniques in the beginning of the 20th century may also have reduced the visibility of the Gravettian in the Lone Valley^43^. Additionally, other factors like population sizes and settlement patterns including intensity of movements and occupation intensity could have led to differences in preservation of archaeological remains in the caves.

Considering the raw material composition of the assemblages (Table 7), constructed from macroscopic analyses, it does not appear that the difference between the Gravettian and Magdalenian sites is greater than the variation within the Magdalenian sites. Differences in site function or duration of site use between the sites seems to be a good explanation for this.

Combining the results of our analysis with typological information on artifacts allows us to draw more detailed conclusions about raw material procurement (Fig. 3). The number of tools for which we can trace long-distance connections is high (Table 7). However, larger items such as cores, refits of reduction sequences and products of core preparation and maintenance demonstrate for the Gravettian and Magdalenian that not only toolkits were transported over long distances^1,2^. The amount of larger transported items can account for extensive planning of raw-material needs^44,45^, however, whether this trend is observable for all sites cannot be conclusively resolved because the number of pieces tested is relatively low. From the sample we analyzed, it seems likely that a combination of both transported personal gear and transported raw material has led to the presence of raw material transported over long distances. From this, we can most plausibly conclude that some of the artifacts transported provide insights into the planning depth of the Gravettian and Magdalenian populations. It also allows us to draw conclusions about the size of their territories^44,45,57^. The transportation of personal gear alone would not allow such a conclusion by itself^45^. In addition, good quality raw material is accessible over large parts of the Swabian Jura, and even in the direct vicinity of the sites, suggesting the need for advanced planning because heavy pieces of raw material were transported over long distances. The territories of hunter-gatherers in the Gravettian and Magdalenian seem to have extended over the entire southern part of Germany from the western side of the Black Forest, the Freiburg basin, to eastern Bavaria, a distance of 300 km. Foragers who lived along the Danube and the Upper Rhine could even have been part of one socio-economic group in the Magdalenian and in the Gravettian. Previously, this was discussed for the Magdalenian^24,46,47^, but our analysis shows that this may already have been the case in the Gravettian. Other evidence pointing eastward for the Magdalenian are painted stones present in the central Swabian Jura (Hohle Fels, Kleine Scheuer) and Eastern Bavaria (Obere Klause)^47,48^. A similar picture emerges from analyses of personal ornaments made from mollusks. These were transported over large distances (e.g. Atlantic, Mediterranean, Paris basin, Mainz basin) and are present in both technocomplexes, although there are differences in the signal intensity^46,47,49–56^.

## Conclusion

Our analysis provides empirically verifiable evidence for reconstructing prehistoric raw-material procurement using infrared spectroscopic measurements. Our case study on the raw material sourcing of Jurassic chert from the central Swabian Jura, Freiburg basin and eastern Bavaria as well as of Tertiary chert from archaeological sites in the Magdalenian and Gravettian in the Swabian Jura shows that the accuracy of macroscopic predictions cannot always be relied upon. In contrast, the results of infrared spectroscopic analyses combined with a neuronal model are better suited for making predictions. Using infrared spectroscopy allows to reconstruct raw-material procurement and settlement system patterns based on empirical and reproducible data. We were able to support previous hypotheses on territories of Magdalenian and Gravettian foraging groups that spanned across 300 km in an east-western direction. Our data show that well established raw-material connections known from the Magdalenian were already in place during the Gravettian prior to the LGM. The presence of tools and cores in the assemblages indicates a great depth of planning for the transport of raw materials for later use. In the future, it would be desirable to also include raw materials from the north and south of the Swabian Alb to gain a more detailed understanding of the raw material procurement and settlement dynamics of Gravettian and Magdalenian foragers and to combine these with data from the procurement of other materials such as shell ornaments.

## Materials and methods

### Geological setting and reference database

**Fig. 5:**
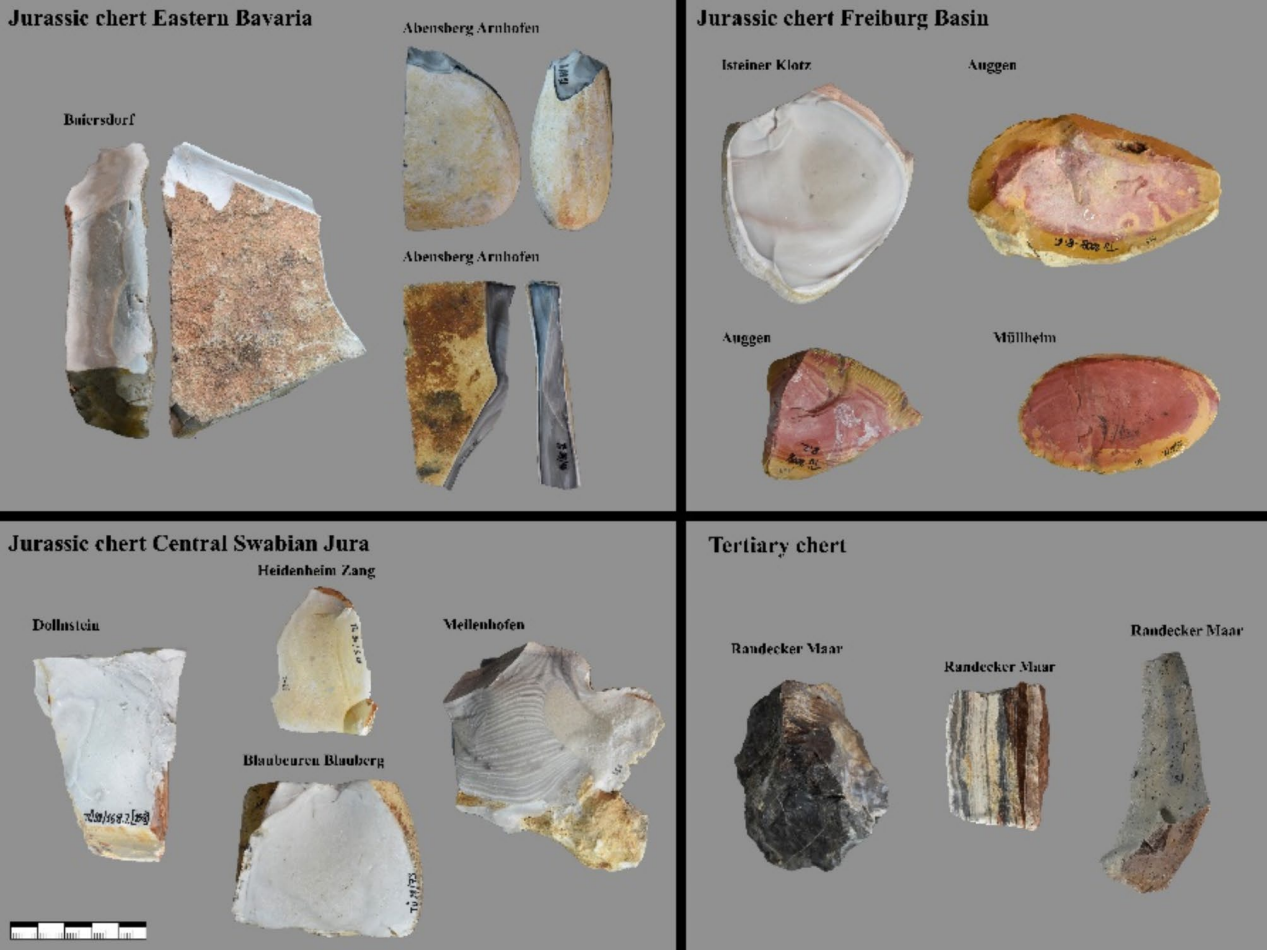
Overview of some of the macroscopic varieties of the raw materials sampled for this study. The Jurassic chert of Eastern Bavaria can occur in tabular form or as nodules and ranges from brown to grey/beige in color. Jurassic chert of the Freiburg basin occurs in nodules ranges in color from red to grey and can have various banding. Jurassic chert of the Central Swabian Jura ranges from brown to grey and appears in different bandings or patterning. Tertiary chert can appear in tabular form or slabs and vary between a blackish color and grey/beige color with different banding or patterning. Photos by B. Schürch.

The Swabian Jura is located north of the Danube and is part of a larger Jurassic massif that stretches from France across Switzerland to Bavaria. To the west, the Black Forrest is a natural border between the Swabian Jura and the Jurassic formations in the Freiburg basin. The Franconian Jura, a continuation of the Swabian Jura, lies to its east. All sampled Jurassic raw material outcrops are located in these three regions (Central Swabian Jura, Eastern Bavaria, Freiburg Basin) (Figs. 1 and 5). We call this raw material *Jurassic chert*. In our reference collection, we included Jurassic cherts sampled in secondary positions that weathered out of their primary layers and were found in overlaying clays^6,8,10^. However, river-transported rocks were not included. Jurassic chert from the Freiburg basin originates from the Kandern-, Korallenkalk- and Nerineenkalk-formations (lower Malm and the uppermost Dogger). Jurassic chert from Eastern Bavaria originates from the Liegende Bankkalk-, Zementmergel- und Hangende Bankkalk-formation (upper Malm). Jurassic chert from the Central Swabian Jura originates from the Liegende Bankkalk-, Zementmergel- und Hangende Bankkalk-formation (upper Malm), the Lacunosamergel-, Untere und Obere Felsenkalk-formation (middle Malm) and the Massenkalk formation (upper middle Malm). We also sampled varieties of Tertiary chert. All of the included samples are freshwater chert. This raw material occurs at three different outcrops: the Randecker Maar (Miocene), Tüllinger Berg (Oligocene) and Steinheim basin (Miocene)^6,8,57,58^. We initially sampled, but then excluded raw materials from the Steinheim basin due to a low signal/noise-ratio in our FTIR-measurements.

In total, our reference database is composed of 1,628 samples from 114 raw material outcrops (see Table 6; n of samples for each region/raw material: Jurassic chert from the Freiburg basin = 403; Jurassic chert from Eastern Bavaria = 240; Jurassic chert from the Central Swabian Jura = 850; Tertiary chert = 135). The 114 outcrops were chosen to cover the four main areas of the study (Freiburg basin, Eastern Bavaria, Central Swabian Jura and Tertiary chert). Areas around archeological sites were sampled intensely to account for the local variability of chert. The reference database was built on the reference collections of the Department of Early Prehistory and Quaternary Ecology collected mainly by Burkert^6^ and Auffermann^38^. Jurassic chert of the central Swabian Jura accounts for the most frequent material class in this database (Table 6). This is due to the large geographical area that it covers. In Eastern Bavaria and the Freiburg basin well-known outcrops were sampled^6,8,57,59^. In most cases several nodules from one outcrop were sampled to account for outcrop variability.

We use color-coding in the tables and maps listing different raw materials for better readability. Jurassic chert of Easter Bavarian is marked blue, Jurassic chert of the Freiburg basin red, Tertiary chert orange; Jurassic chert of the central Swabian Jura grey.

### Infrared spectroscopic analysis and statistical approach

Infrared reflectance spectra were (nondestructively) recorded at the surface of reference samples and artifacts. Recording reflectance spectra is a comparatively time-efficient process, and compiling an extensive reference database is possible. For this purpose, a flat surface with no obvious inclusions was chosen. An Agilent Cary 630 portable spectrometer with a 10° reflectance attachment was used for all analyses. Spectra were acquired between 1300 and 400 cm-1, a resolution of 4 cm-1 and 200 repetitions to improve signal to noise-ratio. The obtained data were first normalized (min-max) over the complete spectral range and then a spectrum of the spectra’s first derivative was calculated. This first derivative data by wavenumber (a set of 243 variables for each sample) was further processed for statistical analysis. Such derivative data are largely independent of band height, mainly reflecting the presence/absence of infrared bands and their shape. The archeological samples were measured three times, after which the data were added and averaged. The mean was then used for the PCA and the following calculations. This was done to include possible variations or zoning in the raw material, which would otherwise not be represented by a single measurement.

Similarities and dissimilarities between sample spectra were analyzed using principal component analysis (PCA). The observed variance between spectra can be related to the sample’s mineralogical composition and variations in quartz crystallography^10,13^. To refine the predictions of the origin of archaeological samples, we used a neural model (with one hidden layer and 6 knots using JMP (JMP-statistical software)) and we conducted a linear discriminant analysis (LDA), both without assigning archeological samples to a group. Linear discriminant analysis was used in a previous study and proved successful^10^. Neural networks were successfully used in several studies before on spectroscopic classification and validation^60–62^. To validate our dataset, we performed an accuracy assessment test on the raw material groups, without including archaeological samples. For this, a random sample set of 30% of the geological references was removed from the groups. These random subsamples were then assigned to the raw material groups by the neural model and the LDA to determine whether they were assigned correctly.

### Theoretical background of the method

**Fig. 6:**
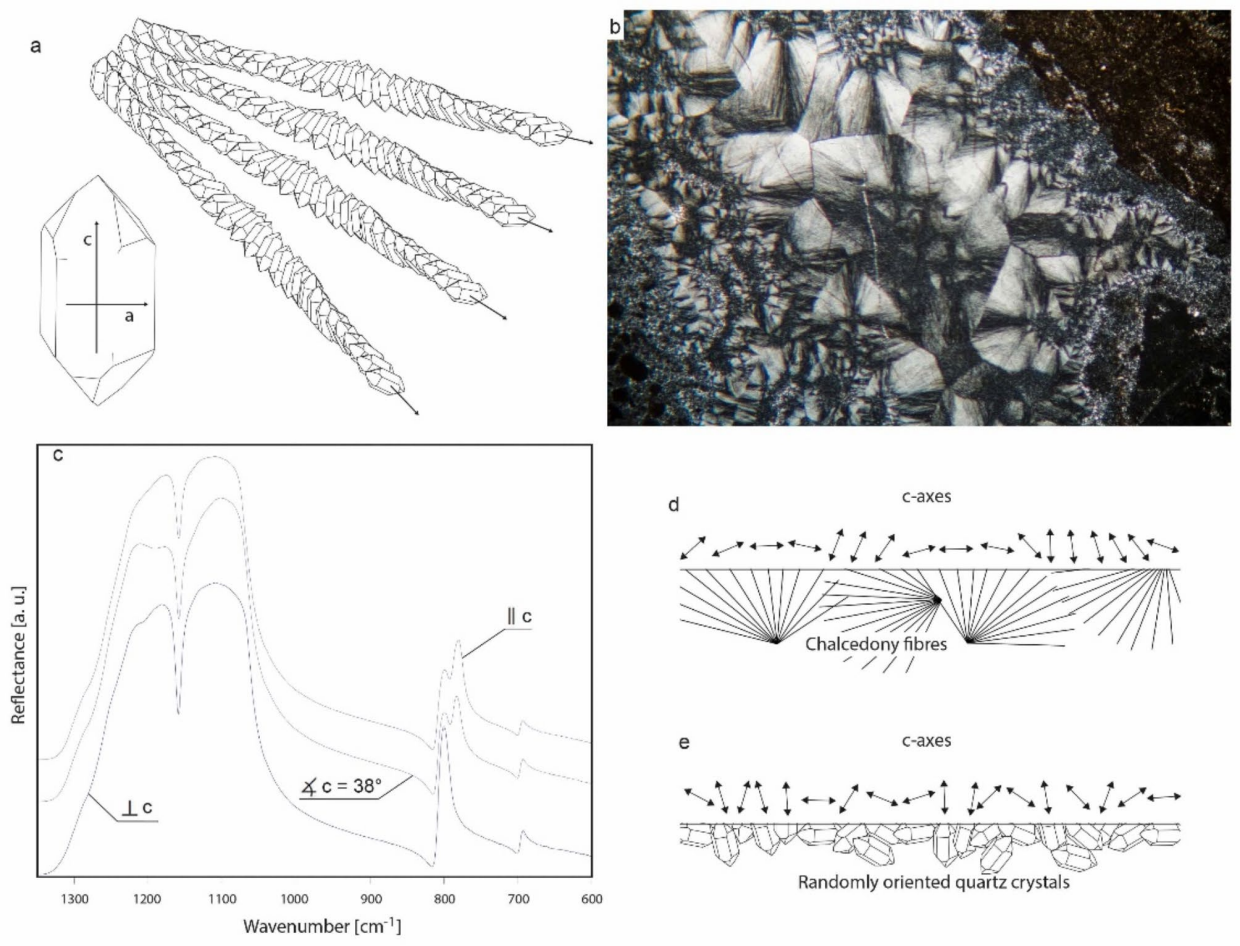
Crystallography of quartz in chert causing differences in infrared reflectance spectra. (**a**) Crystal orientation in length-fast chalcedony, schematic. (**b**) Thin section micrograph of a typical chert sample, showing the presence of fibrous chalcedony and zones where crystal orientation is random (darker zones). (**c**) Reflectance spectra of a quartz crystal acquired with three different inclination angles to the crystal’s c-axis. (**d**) Schematic representation of a section cut normally to the surface of a chert sample consisting of chalcedony fibres. (**e**) Schematic representation of a section cut normally to the surface of a chert sample consisting of micro-quartz. Double-headed arrows schematically show the orientation of the quartz c-axes with respect to the sample surfaces. Photos and graphics by P. Schmidt.

The method we use to test these hypotheses is based on reflectance infrared spectroscopy. It allows for distinguishing groups of samples of silica rocks based on their mineral content and the structure of their quartz crystals (their crystallography). The underlying principle can be summarized as follows. Silica rocks such as flint or chert are mainly composed of quartz crystals present as chalcedony and micro-quartz^63^. Chalcedony has a fibrous texture in thin Sect^64^. It consists of nanometer-sized quartz crystals^65^ that are preferentially oriented, i.e. their *c*-axes have the same inclination with the chalcedony fibre axes^66^. Length-fast chalcedony is the most frequently found type of orientation. Here the crystals’ *c*-axes are perpendicular to the fibre axes. In length-slow chalcedony, the *c*-axes are parallel to the fibre axes, see for example:^67^. Figure 6a is a schematic representation of length-fast chalcedony. The quartz crystals’ *c*-axes are twisted around the fibre axes, for an explanation of this phenomenon see for example:^68^. The typical sample of chert (Fig. 6b) shows fibres of different lengths and darker zones where fibres cannot be recognised at all. Quartz crystals are not oriented preferentially in these zones^69^. Thus, quartz crystals may either be oriented preferentially, as in chalcedony fibres, or randomly, as in zones outside of visible chalcedony fibres. Further, there are samples that contain quartz grains or zones where quartz crystals are sufficiently large to be seen in a microscope. The implications for reflectance infrared spectroscopy are discernible spectral differences. Figure 6c shows three reflectance spectra acquired on a quartz single crystal. Spectra are acquired on different surfaces with different inclinations to the crystal’s *c*-axis. The spectra show differences in the number of bands present, relative band heights, and overall shape of the reflection bands (these phenomena are explained in detail in:^70,71^). Thus, infrared reflectance spectra acquired on chert samples, where quartz crystals are oriented preferentially, are different from spectra of chert, where crystals are oriented randomly. The reason for this is that some crystal orientations, e.g. as in chalcedony fibres, cause alinements of the quartz *c*-axes (with respect to a surface on which reflectance spectra are acquired) to occur more or less often. Figure 6d and e show schematic representations of quartz crystals in two different chert samples. The sample in d is made of chalcedony fibres that are arranged in fans, these are corresponding to spherulites in a 3D model. The sample in 2e is made of micro-quartz crystals that are randomly oriented with respect to the surface. Double-headed arrows indicate the *c*-axis inclination at specific places on the surfaces. From this comparison, it can be seen that similar *c*-axis orientations are present across larger areas on the surface of chalcedony, but not on the surface of randomly oriented quartz. The exact orientation pattern depends on fibre length and the type of chalcedony. Thus, infrared reflectance spectra acquired on chert can be expected to be different, if there are more or fewer zones with chalcedony fibres and longer or shorter fibres. The presence or absence of different types of chalcedony (length-fast of length-slow), hydroxylation (for the resulting spectral differences see:^72^) and crystal size are other factors, which may influence the shape of reflection bands. In other words, different crystallographic properties of quartz in different chert samples lead to different features in the fingerprint region of the quartz infrared spectrum. The presence of phases other than quartz (with an entirely different reflectance spectrum) will lead to further differences in chert spectra. However, even if only quartz is present, infrared spectra can be used to differentiate chert samples based on differences in their crystallography. Thus, the method we use here is based on making statements on the similarity or difference of chert samples in terms of their crystallographic and mineralogical properties.

### Archeological samples

The studied archeological sites represent a significant part of the archeological record of the Gravettian and Magdalenian of Southwestern Germany. Mainly cave sites were included because most open-air sites lack stratigraphic context in the Swabian Jura^73–75^. Most of the sites are cave sites of the Ach and Lone valleys (Fig. 1). The two valleys are about 35 km apart and are both located in the Swabian Alb. Sites in the Ach Valley include Brillenhöhle^29,30,76^, Geißenklösterle^30^, Sirgenstein^77,78^ and Hohle Fels^24,25,47^. Sites in the Lone Valley include Langmahdhalde^35,79^, Vogelherd^80,81^ and Bocksteinhöhle^82^. The two sites located at the northern edge of the Swabian Alb are Kleine Scheuer im Rosenstein^83^ and Burkhardtshöhle^33^. The two sites in the Schmiech Valley are Schmiechenfels^31,84^ and Hohle Fels Hütten^31,84^. An overview of each site is given in the supplementary.

We selected 123 artifacts from 11 sites for which the tested raw materials were identified based on previous studies to test the results of their previous macroscopic analysis (Table 7). The composition of the assemblages and references to the macroscopic analyses are shown in Table 2. It was not always possible to identify the archaeological pieces mentioned in the macroscopic studies we used as the base hypothesis. Therefore, we used illustrated artifacts and the sorting and labelling system of the respective researchers to identify the artifacts. Table 7 shows an overview of the assemblages and the proportions of the tested raw materials. The identification of all raw materials by the referenced researchers can be found in the Supporting Materials Table 1.

## Electronic supplementary material

Below is the link to the electronic supplementary material.

Supplementary Material 1

Supplementary Material 2

Supplementary Material 3

Supplementary Material 4

## Data Availability

The datasets generated during the current study are available in the supplementary Table 3. The predictions for single artifacts generated by the analysis are available in the supplementary Table 2.
