# Supplementary material for: Examining Gravettian and Magdalenian mobility and technological organization with IR spectroscopy
Source: Sci Rep. 2025 Jan 14;15:1897. doi: 10.1038/s41598-024-84302-6 (PMC11730608; doi:10.1038/s41598-024-84302-6)
Supplement: Supplementary file 2 — Supplementary Material 2 [file 41598_2024_84302_MOESM2_ESM.docx]

**Supplementary**

**Bockstein Höhle**
Bockstein Höhle or Bockstein Cave is located in the Lone Valley right next to the well-known Middle Paleolithic site Bocksteinschmiede^1,2^. Bocksteinhöhle was first excavated in 1879 and 1881 by Ludwig Bürger. We included one artifact (ID:49) from the 1883/84 excavations conducted by Bürger and Losch ^1,3,4^. Further excavations took place in 1908 by R. R. Schmidt. From this excavation three other sampled artifacts originate from. The stratigraphy was last revised by Krönneck; she places Layer III of the Schmidt excavation into a Late Magdalenian and Late Aurignacian ^5,6^.

**Brillenhöhle**
Brillenhöhle, located in the Ach Valley, was excavated between 1956 and 1963 under the direction of Gustav Riek and the University of Tübingen ^7^. The stratigraphy of the site includes finds from the Gravettian, Magdalenian, Mesolithic and Neolithic periods ^7,8^. The Magdalenian artifacts sampled are mostly from layer IV (n=25) only one backed artifact from layer III was included. The Gravettian artifacts originate from layer VI (n=2) and VII (n=15). The Magdalenian of the site was analyzed by Lauxmann^9^ and the Gravettian was analyzed by Moreau, Lauxmann and Otte independently^10-12^. The stratigraphy was last revised in 1986 revealing problems with the Riek’s stratigraphy^10^. The broad attribution of the Gravettian (horizon VI and VII) and Magdalenian (mainly horizon IV) can be accepted.

**Burkhardtshöhle**
Burkhardtshöhle, located at the northern rim of the Swabian Alb, was excavated in 1933 and 1934 by Gustav Riek^13,14^. The sampled artefacts originate from this excavation. Horizon V was the only layer containing lithic artifacts (artifacts from this layer are labelled with AH I)^14,15^. In 1973, the site was re-excavated by J. Hahn. Several human skull fragments could be recovered during the reprocessing of the faunal remains of the site, one fragment was directly dated to 12,450 +/-110 uncalBP ^15,16^. Simon classifies the site in the range of the Bølling to older Dryas ^15^.

**Geißenklösterle**Geißenklösterle Cave is located in the Ach Valley and was excavated by Wagner, Hahn and later by Conard between 1973 and 2002 ^11,17,18^. Among the roughly 20.000 lithic artefacts from the Gravettian more than 4.000 were larger than 1cm^11^. The Gravettian of the site dates between 35,000 and 31,000 calBP^11,19^. Refits of lithic artifacts showed that the six initially determined sub layers of the Gravettian represent the palimpsest of one main settlement horizon^17,18^. The tested archeological artifact comes from layer Ip.

**Hohle Fels**Hohle Fels is a cave site located in the Ach Valley. It was first excavated in 1870 by O. Fraas^20,21^. The sampled artifacts from the Gravettian and Magdalenian originate from the more recent excavations by Conard^22-26^. The Magdalenian samples originate from archeological horizons I and IIa. The age of the Magdalenian layers ranges between 16,300 and 14,700 cal BP^24,25^. The Gravettian samples originate from archeological horizons IIb and IIc. The Gravettian dates range between 34,000 and 31,000 cal BP^26^.

**Hohle Fels Hütten**Hohle Fels Hütten is a small rock shelter situated in the Schmiech Valley. The site was excavated in 1906 by R. R. Schmidt ^24,27,28^. The artifacts from the two main horizons (“grey” and “yellow” horizon) point towards a Magdalenian or late Magdalenian age of the deposits^27^. The samples studied come from the yellow and grey horizon, one of them is without context. Later occupation of the sites in the Mesolithic can only be shown by individual artifacts^27^.

**Langmahdhalde**Langmahdhalde is a rock shelter situated in the Lone Valley. The site was first excavated in 2016 with excavations in Middle Paleolithic layers still going on under the directions of N.J. Conard^29-31^. The Magdalenian occupation spans from archeological horizon III to IX. The sampled artifacts originate from AH VI which was directly dated to 14,979 – 14,356 calBP^30^. Until now, the lithic assemblage of the site has not been analyzed completely, therefore the two sampled artifacts^29^ are only a small sample of the raw material variability of the site.

**Kleine Scheuer im Rosenstein**The Kleine Scheuer im Rosenstein is a cave located at the northern rim of the Swabian Alb near Heubach. The site was first excavated in 1912 by H. Maier and later in the same year by R. R. Schmidt^32-34^. The lithic and organic assemblage of layer II and III point towards a Magdalenian age of the layers. One radiocarbon date (12,129-11,832 calBC) from layer II can confirm a late Magdalenian occupation of the site^33^.

**Schmiechenfels**Schmiechenfels is a small rock shelter situated in the Schmiech Valley. Just like Hohle Fels Hütten the site was excavated in 1906 by R.R. Schmidt ^24,27,28^. It was reconstructed that the main occupation of the site took place in the late Magdalenian ^27^. A single arrowhead also indicates a more recent settlement in the Neolithic ^27^.

**Sirgenstein**Sirgenstein Cave is located in the Ach Valley. The site was excavated in 1906 by R. R. Schmidt^28,35,36^. The stratigraphy of the site was recently reevaluated^37^. New radiocarbon evidence showed problems in the stratigraphy but also provided dates for the Gravettian and Magdalenian. The lithic assemblage of layer I can be characterized as Magdalenian, however, the radiocarbon dates also revealed older materials mixed with the Magdalenian^37^. Layer II was confirmed to be of Gravettian age, ranging between 27,700 and 31,600 calBP^37^. The sampled artifacts originate from layer I (Magdalenian) and layer II (Gravettian).

**Vogelherd**Vogelherd Cave is situated in the Lone Valley and was excavated in 1931 by G. Riek ^38^. Riek assigned layer II and III to the Magdalenian ^38^. The backdirt of the site was excavated by the University Tübingen under the direction of N.J.Conard from 2005-2012 and from 2022-2023^39,40^. After a reassessment and redating, layer III was attributed to the Gravettian and layer II was attributed to the Magdalenian ^31,41^. Besides the artifact from layer III and II we included four backed bladelets from the backdirt (geological layer: HL/KS) ^39,42^, these could either come from the Gravettian or the Magdalenian. The Magdalenian of the site dates mainly between 15,100 and 16,200 calBP ^31^. The Gravettian of the site dates between 34,000 and 30,000 calBP ^43^. The stratigraphy of the site is being reassessed by BS. More detailed information on the assemblages of the Magdalenian^31^ and Gravettian will be presented in the near future.

**References**

1 Wetzel, R. *Die Bocksteinschmiede mit dem Bocksteinloch, der Brandplatte und dem Abhang sowie der Bocksteingrotte. Ein Beitrag zur europäischen Urgeschichte des Lonetals und zur geschichtlichen Morphologie des Menschen*. (W. Kohlhammer Verlag, 1958).

2 Wetzel, R. & Bosinski, G. *Die Bocksteinschmiede in Lonetal (Markung Rammingen, Kreis Ulm). Teil I: Text*. 1st. edn, (Müller & Graff, 1969).

3 Bürger, L. Der Bockstein, das Fohlenhaus, der Salzbühl, drei prähistorische Wohnstätten im Lonetal. Ulm, Oberschwaben. *Mitt. Ver. Kunst u. Alterthum Ulm u. Oberschwaben* **3**, 1-29 (1892).

4 Bürger, L. Bockstein - Fohlenhaus - Salzbühl. *Correspondenzblatt der Deutschen Gesellschaft für für Anthropologie, Ethnologie und Urgeschichte* **23**, 107-108 (1892).

5 Krönneck, P. *Die pleistozäne Makrofauna des Bocksteins(Lonetal - Schwäbische Alb) : ein neuer Ansatz zur Rekonstruktion der Paläoumwelt*, (2012).

6 Conard, N. J., Bolus, M., Dutkiewicz, E. & Wolf, S. *Eiszeitarchäologie auf der Schwäbischen Alb die Fundstellen im Ach- und Lonetal und in ihrer Umgebung*. (Kerns, 2015).

7 Riek, G. *Das Paläolithikum der Brillenhöhle bei Blaubeuren (Schwäbische Alb) (Vol. 1, Forschungen und Berichte zur Vor- und Frühgeschichte in Baden-Württemberg, Vol. 4)*. (Müller & Graeff [in Komm.], 1973).

8 Bolus, M. & Conard, N. J. Zur Zeitstellung von Geschossspitzen aus organischen Materialien im späten Mittelpaläolithikum und Aurignacien. *Archäologisches Korrespondenzblatt* **36**, 1-14 (2006).

9 Lauxmann, C. *Die Steinartefakte des Magdalenienzeitlichen Fundhorizontes IV aus der Brillenhöhle bei Blaubeuren unter besonderer Berücksichtigung des Rohmaterials und der Grundproduktion* Magister thesis, University Tübingen, (1988).

10 Lauxmann, C. & Scheer, A. Zusammensetzungen von Steinartefakten. *Fundberichte aus Baden-Württemberg* **11**, 101-132 (1986).

11 Moreau, L. *Geißenklösterle das Gravettien der Schwäbischen Alb im europäischen Kontext*. (Kerns, 2009).

12 Otte, M. *Le Gravettien en Europe Centrale*. (De Tempel, 1981).

13 Riek, G. *Vorgeschichte von Württemberg. Bd. 1 - Kulturbilder aus der Altsteinzeit Württembergs*. (Heine, 1935).

14 Riek, G. Das Federmesserführende Magdalénien der Burhardtshöhle bei Westerheim im Kreis Münsingen (Schwäbische Alb). *Fundberichte aus Schwaben* **15**, 30-42 (1959).

15 Simon, U. *Die Burkhardtshöhle - eine Magdalénienstation am Nordrand der Schwäbischen Alb* Magister thesis, University Tübingen, (1992).

16 Sala, N. & Conard, N. Taphonomic analysis of the hominin remains from Swabian Jura and their implications for the mortuary practices during the Upper Paleolithic. *Quaternary Science Reviews* **150**, 278-300, doi:<https://doi.org/10.1016/j.quascirev.2016.08.018> (2016).

17 Hahn, J. & Owen, L. R. Blade technology in the Aurignacian and Gravettian of Geissenklösterle Cave, Southwest Germany. *World Archaeology* **17**, 61-75, doi:10.1080/00438243.1985.9979950 (1985).

18 Scheer, A. Von der Schichtinterpretation bis zum Besiedlungsmuster – Zusammensetzungen als absoluter Nachweis in *The big puzzle. International Symposium on Refitting Stone Artefacts* (eds Erwin Cziesla, S. Eickhoff, N. Arts, & D. Winter) 623-650 (Holos, 1990).

19 Higham, T. *et al.* Testing models for the beginnings of the Aurignacian and the advent of figurative art and music: the radiocarbon chronology of Geissenklosterle. *J Hum Evol* **62**, 664-676, doi:10.1016/j.jhevol.2012.03.003 (2012).

20 Bolus, M. & Conard, N. J. in *„All der holden Hügel ist keiner mir fremd ...“ Festschrift zum 65. Geburtstag von Claus-Joachim Kind* *Universitätsforschungen zur prähistorischen Archäologie* (eds Michael Baales & Clemens Pasda) Ch. Überblicke zum Paläolithikum und Mesolithikum, 43-66 (Verlag Dr. Rudolf Habelt, 2019).

21 Fraas, O. Resultate der Ausgrabungen im Hohlefels bei Schelklingen. *Jahreshefte des Vereins für vaterländische Naturkunde in Württemberg* **45**, 1-14 (1872).

22 Conard, N. J., Langguth, K. & Uerpmann, H.-P. *Die Ausgrabungen im Gravettien des Hohle Fels bei Schelklingen, Alb-Donau-Kreis*. (Theiss, 2000).

23 Conard, N. J. & Malina, M. Die Ausgrabungen am Hohle Fels bei Schelklingen, Alb-Donau-Kreis. *archäologische Ausgrabungen Baden-Würrtemberg 2006*, 17-20 (2007).

24 Taller, A., Napierala, H., Münzel, S. C. & Conard, N. J. *Das Magdalénien des Hohle Fels chronologische Stellung, lithische Technologie und Funktion der Rückenmesser Die Fauna des Magdalénien vom Hohle Fels*. (Kerns, 2014).

25 Taller, A., Bolus, M. & Conard, N. in *Modes de Contacts et de Déplacements au Paléolothique Eurasiatique Actes du Colloque international de la commission 8 (Paléolithique supérieur) de l´UISPP* (eds Marcel Otte & F. Le Brun-Ricalens) (UISPP, 2014).

26 Taller, A. & Conard, N. J. Das Gravettien der Hohle Fels-Höhle und seine Bedeutung für die kulturelle Evolution des europäischen Jungpaläolithikums. *Quartär* **63**, 89-123 (2016).

27 Karle, I. *Das Magdalénien im Schmiechtal* Magister thesis, University Tübingen, (1997).

28 Schmidt, R. R. *Die diluviale Vorzeit Deutschlands. with contributions by E. Koken & A. Schliz*. (E. Schweizerbartsche Verlagsbuchhandlung, 1912).

29 Conard, N. J., Janas, A. & Zeidi, M. Ausgrabungen an der Langmahdhalde liefern weitere Einblicke in magdalénienzeitliche Lebensweisen auf der Schwäbischen Alb. *Archäologische Ausgrabungen Baden-Würrtemberg* **2018** (2019).

30 Wong, G. L., Drucker, D. G., Starkovich, B. M. & Conard, N. J. Latest Pleistocene paleoenvironmental reconstructions from the Swabian Jura, southwestern Germany: Evidence from stable isotope analysis and micromammal remains. *Palaeogeography, Palaeoclimatology, Palaeoecology* **540**, 109527, doi:<https://doi.org/10.1016/j.palaeo.2019.109527> (2020).

31 Schürch, B., Wong, G. L., Luzi, E. & Conard, N. J. New evidence for an earlier Magdalenian presence in the Lone Valley of southwest Germany. *Journal of Archaeological Science* (accepted).

32 Maier, H. Die altsteinzeitliche Wohnhöhle „Kleine Scheuer“ im Rosenstein. *Zeitschrift für Deutsche Vorgeschichte* **28**, 235-252 (1936).

33 Wettengl, S. Das Magdalénien um Heubach – Die Kleine Scheuer, der Sand und die Schlattäcker bei Waldstetten in *Das Magdalénien im Südwesten Deutschlands, im Elsass und in der Schweiz* (ed H. Floss) (Kerns Verlag, 2019).

34 Riek, G. *Kulturbilder aus der Altsteinzeit Württembergs*. (Heine, 1935).

35 Schmidt, R. R. Der Sirgenstein und die eiszeitlichen Kulturepochen Schwabens. *Fundberichte aus Schwaben* **15**, 2-7 (1907).

36 Schmidt, R. R. *Der Sirgenstein und die diluvialen Kulturstätten Württembergs*. (E. Schweizerbart´sche Verlagsbuchhandlung, 1910).

37 Bertacchi, A., Starkovich, B. M. & Conard, N. J. The Zooarchaeology of Sirgenstein Cave: A Middle and Upper Paleolithic site in the Swabian Jura, SW Germany. *Journal of Paleolithic Archaeology* **4**, 7, doi:10.1007/s41982-021-00075-8 (2021).

38 Riek, G. *Die Eiszeitjägerstation am Vogelherd im Lonetal. - Die Kulturen*. (Kabitzsch, 1934).

39 Conard, N. J., Zeidi, M. & Janas, A. Abschließender Bericht über die Nachgrabung am Vogelherd und die Sondage in der Wolftalhöhle. *Archäologische Ausgrabungen Baden-Würrtemberg 2015*, 66-72 (2016).

40 Conard, N. J., Zeidi, M. & Janas, A. Neue Ausgrabungen am Vogelherd im Lonetal. *Archäologische Ausgrabungen Baden-Würrtemberg* **2022**, 57-59 (2023).

41 Schürch, B. & Conard, N. J. Reassessing the cultural stratigraphy of Vogelherd Cave and the settlement history of the Lone Valley of SW Germany in *European Society for the study of Human Evolution,* abstract (Tübingen, 2022).

42 Conard, N. & Zeidi, M. Ausgrabungen in der Fetzershaldenhöhle und der Lindenhöhle im Lonetal sowie neue Funde aus dem Vogelherd. *Archäologische Ausgrabungen Baden-Württemberg 2013*, 63-67 (2014).

43 Conard, N., Niven, L., Mueller, K. & Stuart, A. The Chronostratigraphy of the Upper Paleolithic Deposits at Vogelherd. *Mitteilungen der Gesellschaft für Urgeschichte* **12**, 73-86 (2003).

**Supplementary Table 1.** Overview of the Archeological sites with the corresponding layers, technocomplexes, sources of the macroscopic determinations, assemblage size and percentages of all macroscopically determined raw materials in the archeological assemblages.

**Supplementary Table 2.** Predictions of the neural model and the LDA
